# A Survey of Automatic Facial Micro-Expression Analysis: Databases, Methods, and Challenges

**DOI:** 10.3389/fpsyg.2018.01128

**Published:** 2018-07-10

**Authors:** Yee-Hui Oh, John See, Anh Cat Le Ngo, Raphael C. -W. Phan, Vishnu M. Baskaran

**Affiliations:** ^1^Faculty of Engineering, Multimedia University Cyberjaya, Malaysia; ^2^Faculty of Computing and Informatics, Multimedia University Cyberjaya, Malaysia; ^3^School of Psychology, University of Nottingham Nottingham, United Kingdom; ^4^Research Institute for Digital Security, Multimedia University Cyberjaya, Malaysia; ^5^School of Information Technology, Monash University Malaysia Bandar Sunway, Malaysia

**Keywords:** facial micro-expressions, subtle emotions, survey, spotting, recognition, databases, spontaneous, expressions

## Abstract

Over the last few years, automatic facial micro-expression analysis has garnered increasing attention from experts across different disciplines because of its potential applications in various fields such as clinical diagnosis, forensic investigation and security systems. Advances in computer algorithms and video acquisition technology have rendered machine analysis of facial micro-expressions possible today, in contrast to decades ago when it was primarily the domain of psychiatrists where analysis was largely manual. Indeed, although the study of facial micro-expressions is a well-established field in psychology, it is still relatively new from the computational perspective with many interesting problems. In this survey, we present a comprehensive review of state-of-the-art databases and methods for micro-expressions spotting and recognition. Individual stages involved in the automation of these tasks are also described and reviewed at length. In addition, we also deliberate on the challenges and future directions in this growing field of automatic facial micro-expression analysis.

## 1. Introduction

In 1969, Ekman and Friesen (1969) spotted a quick full-face emotional expression in a filmed interview which revealed a strong negative feeling a psychiatric patient was trying to hide from her psychiatrist in order to convince that she was no longer suicidal. When the interview video was played in slow motion, it was found that the patient was showing a very brief sad face that lasted only for two frames (1/12s) followed by a longer-duration false smile. This type of facial expressions is called micro-expressions (MEs) and they were actually first discovered by Haggard and Isaacs (1966) 3 years before the event happened. In their study, Haggard and Isaacs discovered these micromomentary expressions while scanning motion picture films of psychotherapy hours, searching for indications of non-verbal communication between patient and therapist.

MEs are very brief, subtle, and involuntary facial expressions which normally occur when a person either deliberately or unconsciously conceals his or her genuine emotions (Ekman and Friesen, 1969; Ekman, 2009b). Compared to ordinary facial expressions or macro-expressions, MEs usually last for a very short duration which is between 1/25 and 1/5 of a second (Ekman, 2009b). Recent research by Yan et al. (2013a) suggest that the generally accepted upper limit duration of a micro-expression is within 0.5s. Besides short duration, MEs also have other significant characteristics such as low intensity and fragmental facial action units where only part of the action units of full-stretched facial expressions are presented (Porter and Ten Brinke, 2008; Yan et al., 2013a). Due to these three characteristics of the MEs, it is difficult for human beings to perceive micro-expressions with the naked eye.

In spite of these challenges, new psychological studies of MEs and computational methods to spot and recognize MEs have been gaining more attention lately because of its potential applications in many fields, i.e., clinical diagnosis, business negotiation, forensic investigation, and security systems (Ekman, 2009a; Frank et al., 2009a; Weinberger, 2010). One of the very first efforts to improve the human ability at recognizing MEs was conducted by Ekman where he developed the Micro-Expression Training Tool (METT) to train people to recognize seven categories of MEs (Ekman, 2002). However, it was found in Frank et al. (2009b) that the performance of detecting MEs by undergraduate students only reached at most 40% with the help of METT while unaided U.S. coast guards performed not more than 50% at best. Thus, an automatic ME recognition system is in great need in order to help detect MEs such as those exhibited in lies and dangerous behaviors, especially with the modern advancements in computational power and parallel multi-core functionalities. These have enabled researchers to perform video processing operations that used to be infeasible decades ago, increasing the capability of computer-based understanding of videos in solving different real-life vision problems. Correspondingly, in recent years researchers have moved beyond psychology to using computer vision and video processing techniques to automate the task of recognizing MEs.

Although normal facial expression recognition is now considered a well-established and popular research topic with many good algorithms developed (Zeng et al., 2009; Bettadapura, 2012; Sariyanidi et al., 2015) with accuracies exceeding 90%, in contrast the automatic recognition of MEs from videos is still a relatively new research field with many challenges. One of the challenges faced by this field is spotting the ME of a person accurately from a video sequence. As a ME is subtle and short, spotting of MEs is not an easy task. Furthermore, spotting of MEs becomes harder if the video clip consists of spontaneous facial expressions and unrelated facial movements, i.e., eye-blinking, opening and closing of mouth, etc. On the other hand, other challenges of ME recognition include inadequate features for recognizing MEs due to its low change in intensity and lack of complete, spontaneous and dynamic ME databases.

In the past few years, there have been some noteworthy advances in the field of automatic ME spotting and recognition. However, there is currently no comprehensive review to chart the emergence of this field and summarize the development of techniques introduced to solve these tasks. In this survey paper, we first discuss the existing ME corpora. In our perspective, automatic ME analysis involves two major tasks, namely, ME spotting and ME recognition. ME spotting focuses on finding the occurrence of MEs in a video sequence while ME recognition involves assigning an emotion class label to an ME sequence. For both tasks, we look into the range of methods that have been proposed and applied to various stages of these tasks. Lastly, we discuss the challenges in ME recognition and suggest some potential future directions.

## 2. Micro-expression databases

The prerequisite of developing any automatic ME recognition system is having enough labeled affective data. As ME research in computer vision has only gained attention in the past few years, the number of publicly available spontaneous ME databases is still relatively low. Table 1 gives the summary of all available ME databases to date, including both posed and spontaneous ME databases. The key difference between posed and spontaneous MEs is in the relevance between expressed facial movement and underlying emotional state. For posed MEs, facial expressions are deliberately shown and irrelevant to the present emotion of senders, therefore not really helpful for the recognition of real subtle emotions. Meanwhile, spontaneous MEs are the unmodulated facial expressions that are congruent with an underlying emotional state (Hess and Kleck, 1990). Due to the nature of the posed and spontaneous MEs, the techniques for inducing facial expressions (for purpose of constructing a database) are contrasting. For the case of posed MEs, subjects are usually asked to relive an emotional experience (or even watching example videos containing MEs prior to the recording session) and perform the expression as well as possible. However, eliciting spontaneous MEs is more challenging as the subjects have to be involved emotionally. Usually, emotionally evocative video episodes are used to induce the genuine emotional state of subjects, and the subjects have to attempt to suppress their true emotions or risk getting penalized.

**Table 1:** Micro-expression databases.

**Databases**	**Subset**	**Subjects**	**Samples**	**Frames per sec**	Type^*^	**FACS coded**	**Emotion classes**	**Expression**	**Frame annotations**
USF-HD		–	100	30	P	No	6	Macro/micro	–
Polikovsky's		10	42	200	P	No	6	Micro	–
YorkDDT		9	18	25	S	No	2	Micro	–
Silesian deception^†^		101	101	100	S	No	–	Macro/micro	Eye closures, gaze aversion, micro-tensions
SMIC-sub		6	77	100	S	No	3	Micro	–
SMIC	HS	16	164	100	S	No	3	Micro	–
	VIS	8	71	25	S	No	3		
	NIR	8	71	25	S	No	3		
	E-HS	16	157	100	S	No	3	Micro	Onset,offset
	E-VIS	8	71	25	S	No	3		
	E-NIR	8	71	25	S	No	3		
CASME		19	195	60	S	Yes	7	Micro	Onset,offset,apex
CASME II		26	247	200	S	Yes	5	Micro	Onset,offset,apex
CAS(ME)^2^	Part A	22	87	30	S	Yes	4	Macro/Micro	Onset,offset,apex
	Part B	22	57	30	S	Yes	4		
SAMM		32	159	200	S	Yes	7^‡^	Macro/micro	Onset,offset,apex
MEVIEW		16	31	25	S	Yes	5^§^	macro/micro	onset,offset

* *P/S, Posed/Spontaneous*.

† *Not all samples contain micro-expressions and only a total of 183 occurrences of “micro-tensions” were annotated. No emotion classes were available*.

‡ *Seven objective classes are also provided (Davison et al., 2017)*.

§ *Set of emotions are atypical (contempt, surprise, fear, anger, happy), likely in the context of environment. Some sample clips involve person speaking, or only have AUs marked with no emotions observed*.

According to Ekman and Friesen (1969) and Ekman (2009a), MEs are involuntary which could not be created intentionally. Thus, posed MEs usually do not exhibit the characteristics (i.e., the appearance and timing) of spontaneously occurring MEs (Porter and Ten Brinke, 2008; Yan et al., 2013a). The early USD-HD (Shreve et al., 2011) and Polikovsky's (Polikovsky et al., 2009) databases consist of posed MEs rather than spontaneous ones; hence they do not present likely scenarios encountered in real life. In addition, the occurrence duration of their micro-expressions (i.e., 2/3 s) exceeds the generally acceptable duration of MEs (i.e., 1/2 s). To have a more ecological validity, research interest then shifted to spontaneous ME databases. Several groups have developed a few spontaneous MEs databases to aid researchers in the development of automatic ME spotting and recognition algorithms. To elicit MEs spontaneously, participants are induced by watching emotional video clips to experience a high arousal, aided by an incentive (or penalty) to motivate the disguise of emotions. However, due to the challenging process of eliciting these spontaneous MEs, the number of samples collected for these ME databases is still limited.

Table 1 summarizes the known ME databases in the literature, which were elicited through both posed and spontaneous means. The YorkDDT (Warren et al., 2009) is the smallest and oldest database, with spontaneous MEs that also include other irrelevant head and face movements. The Silesian Deception database (Radlak et al., 2015) was created for the purpose of recognizing deception through facial cues. This database is annotated with eye closures, gaze aversion, and micro-expression, or “micro-tensions,” a phrase used by the authors to indicate the occurrence of rapid facial muscle contraction as opposed to having an emotion category. This dataset is not commonly used in spotting and recognition literature as it does not involve expressions *per se*; its inception primarily for the purpose of automatic deception recognition.

**Figure 1:**
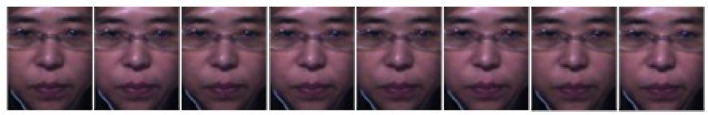
Sample frames from a “Surprise” sequence (Subject 1) in SMIC. Images reproduced from the database with permission from Li et al. (2013).

The SMIC-sub (Pfister et al., 2011) database presents a better set of spontaneous ME samples in terms of frame rate and database size. Nevertheless, it was further extended to the SMIC database (Li et al., 2013) with the inclusion of more ME samples and multiple recordings using different cameras types: high speed (HS), normal visual (VIS), and near-infrared (NIS). However, the SMIC-sub and SMIC databases do not provide Action Unit (AU) (i.e., facial components that are defined by FACS to taxonomize facial expressions) labels and the emotion classes were only based on participants' self-reports. Sample frames from SMIC are shown in Figure 1.

**Figure 2:**
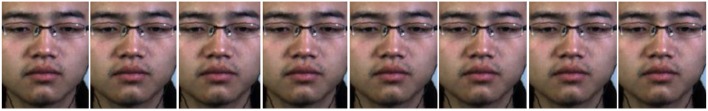
Sample frames from a “Happiness” sequence (Subject 6) in CASME II. Images reproduced from the database with permission from Yan et al. (2014a).

The CASME dataset (Yan et al., 2013b) provides a more comprehensive spontaneous ME database with a larger amount of MEs as compared to SMIC. However, some videos are extremely short, i.e., < 0.2 s, hence poses some difficulty for ME spotting. Besides, CASME samples were captured only at 60 *fps*. An improved version of it, known as CASME II was established to address these inadequacies. The CASME II database (Yan et al., 2014a) is the largest and most widely used database to date (247 videos, sample frames in Figure 2) with samples recorded using high frame-rate cameras (200 *fps*).

**Figure 3:**
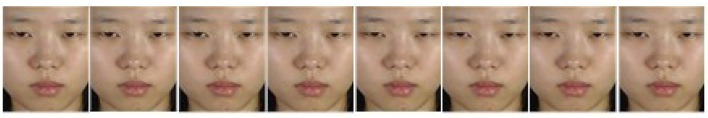
Sample frames from a “Disgust” sequence (Subject 15) in CAS(ME)^2^. Images reproduced from the database (©Xiaolan Fu) with permission from Qu et al. (2017).

To facilitate the development of algorithms for ME spotting, extended versions of SMIC (SMIC-E-HS, SMIC-E-VIS, SMIC-E-NIR), CAS(ME)^2^ (Qu et al., 2017), and SAMM (Davison et al., 2016a) databases were developed. In SMIC-E databases, long video clips that contain some additional non-micro frames before and after the labeled micro frames were included as well. The CAS(ME)^2^ database (with samples given in Figure 3) is separated into two parts: Part A contains both spontaneous macro-expressions and MEs in long videos; and Part B includes cropped expression samples with frame from onset to offset. However, CAS(ME)^2^ is recorded using a low frame-rate (25 *fps*) camera due to the need to capture both macro- and micro-expressions.

**Figure 4:**
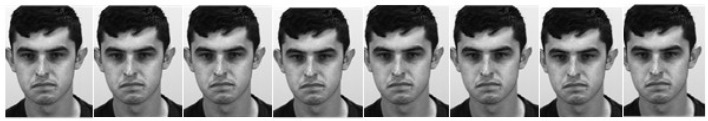
Sample frames from a sequence (Subject 6) in SAMM that contains micro-movements. Images reproduced from the database with permission from Davison et al. (2016a).

In the SAMM database (with samples shown in Figure 4), all micro-movements are treated objectively, without inferring the emotional context after each experimental stimulus. Emotion classes are then labeled by trained experts later. In addition, about 200 neutral frames are included before and after the occurrence of the micro-movement, which makes spotting feasible. The SAMM is arguably the most culturally diverse database among all of them. In short, the SMIC, CASME II, CAS(ME)^2^, and SAMM are considered the state-of-the-art databases for ME spotting and recognition that should be widely adopted for research.

**Figure 5:**
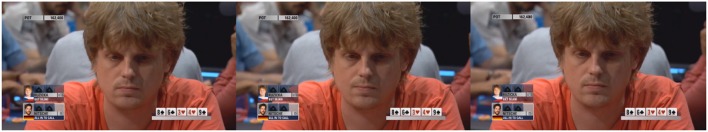
Sample frames from a “Contempt” sequence in MEVIEW that contains micro-movements marked with AU L12. Images reproduced from the database (Husak et al., 2017) under Fair Use.

The need for data acquired from more unconstrained “in-the-wild” situations have compelled further efforts to provide more naturalistic high-stake scenarios. The MEVIEW dataset (Husak et al., 2017) was constructed by collecting mostly poker game videos downloaded from YouTube with a close-up of the player's face (samples frames in Figure 5). Poker games are highly competitive with players often try to conceal or fake their true emotions, which facilitates likely occurrences of MEs. With the camera view switching often, the entire shot with a single face in video (averaging 3s in duration) was taken. An METT-trained annotator labeled the onset and offset frames of the ME with FACS coding and emotion types. A total of 31 videos with 16 individuals were collected.

## 3. Spotting of facial micro-expressions

**Figure 6:**
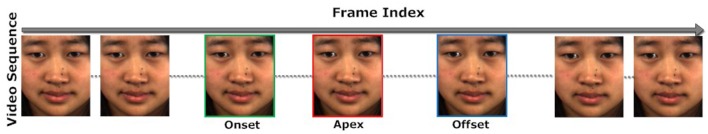
A video sequence depicting the order in which onset, apex and offset frames occur. Sample frames are from a “Happiness” sequence (Subject 2) in CASME II. Images reproduced from the database with permission from Yan et al. (2014a).

Automatic ME analysis involves two tasks: ME spotting and ME recognition. Facial ME spotting refers to the problem of automatically detecting the temporal interval of a micro-movement in a sequence of video frames; and ME recognition is the classification task to identify the ME involved in the video samples. In a complete facial ME recognition system, accurately and precisely identifying frames containing facial micro-movements (which contribute to facial MEs) in a video is a prerequisite for high-level facial analysis (i.e., facial ME recognition). Thus, the automatic facial expression spotting frameworks are developed to automatically search the temporal dynamics of MEs in streaming videos. Temporal dynamics refer to the motions of facial MEs that involve onset(start), apex(peak), offset(end), and neutral phases. Figure 6 shows a sample sequence depicting these phases. According to the work by Valstar and Pantic (2012), the onset phase is the moment where muscles are contracting and appearance of facial changes grows stronger; the apex phase is the moment where the expression peaks (the most obvious); and the offset phase is the instance where the muscles are relaxing and the face returns to its neutral appearance (little or no activation of facial muscles). Typically a facial motion shifts through the sequence of neutral-onset-apex-offset-neutral, but other combinations such as multiple apices are also possible.

In general, a facial ME spotting framework consists of a few stages: the pre-processing, feature description, and lastly the detection of the facial micro-expressions. The details of each of the stages will be further discussed in the following sections.

### 3.1. Pre-processing

In facial ME spotting, the general pre-processing steps include facial landmark detection, facial landmark tracking, face registration, face masking, and face region retrieval. Table 2 shows a summary of existing pre-processing techniques that are applied in facial ME spotting.

**Table 2:** A survey of pre-processing techniques applied in facial micro-expression spotting.

**Work**	**Landmark detection**	**Landmark tracking**	**Face registration**	**Masking**	**Face regions**
Polikovsky et al., 2009	Manual	–	–	–	12 ROIs
Shreve et al., 2009	–	–	–	–	3 ROIs
Wu et al., 2011	–	–	–	–	Whole face
Shreve et al., 2011	–	–	Face alignment	Eyes, nose	8 ROIs
				and mouth	
Polikovsky and Kameda, 2013	Manual	APF	–	–	12 ROIs
Shreve et al., 2014	SCMS	–	–	Eyes and mouth	4 Parts
Moilanen et al., 2014	Manual	KLT	Face alignment	–	6 × 6 blocks
Davison et al., 2015	Face++	–	Affine transform	–	5 × 5 blocks
Patel et al., 2015	DRMF	OF	–	–	49 ROIs
Liong et al., 2015	DRMF	–		–	3 ROIs
Wang et al., 2016a	DRMF	–	Non-reflective similarity transformation	–	6 × 6 blocks
Liong et al., 2016c	DRMF	–	–	Eyes	3 ROIs
Xia et al., 2016	ASM	–	Procrutes analysis	–	Whole face
Liong et al., 2016b	DRMF	–	–	–	3 ROIs
Davison et al., 2016a	Face++	–	Affine transform	–	4 × 4, 5 × 5 blocks
Davison et al., 2016b	Face++	–	2D-DFT and Piecewise affine warping	Binary masking	26 ROIs
Yan and Chen, 2017	CLM	–	–	–	16 ROIs
Li et al., 2017	Manual	KLT	–	–	6 × 6 blocks
Ma et al., 2017	CLNF from OpenFace	KLT	–	–	5 ROIs
Qu et al., 2017	ASM	–	LWM	–	Various block sizes
Duque et al., 2018	AAM	KLT	–	–	5 ROIs

#### 3.1.1. Facial landmark detection and tracking

Facial landmark detection is the first most important step in the spotting framework to locate the facial points on the facial images. In the field of MEs, two ways of locating the facial points are applied: the manual method and automatic facial landmark detection method. In an early work on facial micro-movement spotting (Polikovsky et al., 2009), facial landmarks are manually selected only at the first frame, and fixed in the consecutive frames as they assumed that the examined frontal faces are located relatively in the same location. In their later work (Polikovsky and Kameda, 2013), a tracking algorithm is applied to track the facial points that had been manually detected at the first frame throughout the whole sequence. To prevent the hassle of manually detecting the facial points, majority of the recent works (Davison et al., 2015, 2016a,b; Liong et al., 2015, 2016b,c; Wang et al., 2016a; Xia et al., 2016) opt to apply automatic facial landmark detection. Instead of running the detection for the whole sequence of facial images, the facial points are only detected at the first frame and fixed in the consecutive frames with the assumption that these points will only change minimally due to the subtleness of MEs.

To the best of our knowledge, the facial landmark detection techniques that are commonly employed for facial ME spotting are promoted Active Shape Model (ASM) (Milborrow and Nicolls, 2014), Discriminative Response Maps Fitting (DRMF) (Asthana et al., 2013), Subspace Constrained Mean-Shifts (SCMS) (Saragih et al., 2009), Face++ automatic facial point detector (Megvii, 2013), and Constraint Local Model (CLM) (Cristinacce and Cootes, 2006). In fact, the promoted ASM, DRMF, and CLM are the notable examples of part based facial deformable models. Facial deformable models can be roughly separated into two main categories: holistic (generative) models and part based (discriminative) models. The former applies holistic texture-based facial representation for the generic face fitting scenario; and the latter uses the local image patches around the landmark points for the face fitting scenario. Although the holistic-based approaches are able to achieve impressive registration quality, these representations unfaithfully locate facial landmarks in unseen images, when target individuals are not included in the training set. As a result, part based models which circumvent several drawbacks of holistic-based methods, are more frequently employed in locating facial landmarks in recent years (Asthana et al., 2013). The promoted ASM, DRMF, and CLM are from part based deformable models, however their mechanisms are different. The ASM applies shape constraints and searches locally for each feature point's best location; whereas DRMF learns the variation in appearance on a set of template regions surrounding individual features and updates the shape model accordingly; as for CLM, it learns a model of shape and texture variation from a template (similar to active appearance models), but the texture is sampled in patches around individual feature points. In short, the DRMF is computationally lighter than its counterparts.

Part based approaches mainly rely on optimization strategies to approximate the responses map through simple parametric representations. However, some ambiguities still result due to the landmark's small support region and imperfect detectors. In order to address these ambiguities, SCMS which employs Kernel Density Estimator (KDE) to form a non-parametric representation of response maps was proposed. To maximize over the KDE, the mean-shift algorithm was applied. Despite the progress in automatic facial landmark detection, these approaches are still not considerably robust toward “in-the-wild” scenarios, where large out-of-plane tilting and occlusion might exist. The Face++ automatic facial point detector was developed by Megvii (2013) to address such challenges. It employs a coarse-to-fine pipeline with neural network and sequential regression, and it claims to be robust against influences such as partial occlusions and improper head pose up to 90° tilt angle. The efficacy of the method (Zhang et al., 2014) has been tested on the 300-W dataset (Sagonas et al., 2013) (which focuses on facial landmark detection in real-world facial images captured in-the-wild), yielding the highest accuracy among the several recent state-of-the-arts including DRMF.

In ME spotting research, very few works applied tracking to the landmark points. This could be due to the sufficiency of landmark detection algorithms used (since MEs movements are very minute) or that general assumptions have been made to fix the location of the detected landmarks points. The two tracking algorithms that were reportedly used in a few facial ME spotting works (Polikovsky and Kameda, 2013; Moilanen et al., 2014; Li et al., 2017) are Auxiliary Particle Filtering (APF) (Pitt and Shephard, 1999) and Kanade-Lucas-Tomasi (KTL) algorithm (Tomasi and Kanade, 1991).

#### 3.1.2. Face registration

Image registration is the process of aligning two images—the reference and sensed images, geometrically. In the facial ME spotting pipeline, registration techniques are applied onto the faces to remove large head translations and rotations that might affect the spotting task. Generally, registration techniques can be separated into two major categories: area-based and feature-based approaches. In each of the approaches, either global mapping functions or local mapping functions are applied to transform the the sensed image to be as close as the reference image.

For area-based (a.k.a. template matching or correlation-like) methods, windows of predefined size or even entire images are utilized for the correspondence estimation during the registration. This approach bypasses the need for landmark points, albeit some restriction to only shift and small rotations between the images (Zitova and Flusser, 2003). In the work by Davison et al. (2016b), a 2D-Discrete Fourier Transform (2D-DFT) was used to achieve face registration. This method calculates the cross-correlation of the sensed and reference images before finding the peak, which in turn is used to find the translation between the sensed and reference images. Then, the process of warping to a new image is performed by piece-wise affine (PWA) warping.

For feature-based approach to face registration, salient structures which include region features, line features and point features are exploited to find the pairwise correspondence between the sensed and reference images. Thus, feature-based approach are usually applied when the local structures are more significant than the information carried by the image intensities. In some ME works (Shreve et al., 2011; Moilanen et al., 2014; Li et al., 2017), the centroid of the two detected eyes are selected as the distinctive point (also called control points) and exploited for face registration by using affine transform or non-reflective similarity transform. The consequence of such simplicity entails their inability to handle deformations locally. A number of works (Li et al., 2017; Xu et al., 2017) employed Local Weighted Mean (LWM) (Goshtasby, 1988) which seeks to find a 2-D transformation matrix using 68 facial landmark points of a model face (typically from the first frame). In another work by Xia et al. (2016), Procrustes analysis is applied to align the detected landmark points in frames. It determines a linear transformation (such as translation, reflection, orthogonal rotation, and scaling) of the points in sensed images to best conform them to points in the reference image. Procrustes analysis has several advantages: low complexity for easy implementation and it is practical for similar object alignment (Ross, 2004). However, it requires a one-to-one landmark correspondence and the convergence of means is not guaranteed.

Instead of using mapping functions to map the sensed images to the reference images, a few studies (Shreve et al., 2011; Moilanen et al., 2014; Li et al., 2017) correct the mis-alignment by rotating the faces according to the angle between the pair of lines that join the centroids of the two detected eyes to the horizontal line. In this mechanism, errors can creep in if the face contours of the sensed and reference face images are not consistent with one another, or that the subject's face is not entirely symmetrical to begin with.

Due to the diversity of face images with various types of degradations to be registered, it is challenging to fix a standard method that is applicable to all conditions. Thus, the choice of registration method should correspond to the assumed geometric deformation of the sensed face image.

#### 3.1.3. Masking

In the facial ME spotting task, a masking step can be applied onto the face images to remove noise caused by undesired facial motions that might affect the performance of the spotting task. In the work by Shreve et al. (2011), a static mask (“T”-shaped) was applied on the face images to remove the middle part of the face that includes the eyes, nose, and mouth regions. Eye regions were removed to avoid the noise caused by eye cascades and blinking (which is not considered a facial micro-expression); the nose region is masked as it is typically rigid, which might not reveal much significant information even with it; and mouth region is excluded since opening and closing of the mouth introduces undesired large motion. It is arguable if too much meaningful information may have been removed from the face area in the masking steps introduced in Shreve et al. (2011) and Shreve et al. (2014), as the two most expressive facial parts (in the context of MEs) are actually located near the corner of the eyebrow and mouth areas. Hence, some control is required to prevent excluding too much meaningful information. Typically, specific landmark points around these two areas are used as reference or boundary points in the masking process.

In the work by Liong et al. (2016c), the eye regions are masked to reduce false spotting of the apex frame from long videos. They observed that eye blinking motion is significantly more intense than that of micro-expression motion, thus masking is necessary. To overcome potential inaccurate landmark detection, a 15-pixel margin was added to extend the masked region. Meanwhile, Davison et al. (2016b) applied a binary mask to obtain 26 FACS-based facial regions that include the eyebrows, forehead, cheeks, corners around eyes, mouth, regions around mouth, and etc. The regions are useful for the spotting task as each of these regions contain a single or a group of AUs, which will be triggered when the ME occurs. It is also worth mentioning that a majority of works in the literature still do not include a masking pre-processing step.

#### 3.1.4. Face region retrieval

From psychological findings on concealed emotions (Porter and Ten Brinke, 2008), it was revealed that facial micro-expression analysis should be done on the upper and lower halves of the face separately instead of considering the entire face. This finding substantiated an earlier work (Rothwell et al., 2006), whereby ME recognition was also performed on the segmented upper and lower parts of the face. Duan et al. (2016) later showed that the eye region is much more salient than the whole face or mouth region for recognizing micro-expressions, in particular happy and disgust expressions. Prior knowledge from these works encourage splitting of the face into important regions for automatic facial micro-expression spotting.

In the pioneering work of spotting facial MEs (Shreve et al., 2009), the face was segmented into three regions: the upper part (which includes the forehead), middle part (which includes the nose and cheeks), and the lower part (which include the mouth); and each was analyzed as individual temporal sequences. In their later work (Shreve et al., 2011), the face image is further segmented into eight regions: forehead, left and right of the eye, left and right of cheek, left and right of mouth and chin. Each of the segments is analyzed separately in sequence. With the more localized segments, tiny changes in certain temporal segments could be observed. However, unrelated edged features such as hair, neck, and edge of the face that are present in the localized segments might induce noise and thus affect the extracted features. Instead of splitting the face images into few segments, Shreve et al. (2014) suggested to separate the face images into four quadrants, and each of the quadrant is analyzed individually in the temporal domain. The reason behind this is because of the constraint on locality as facial micro-expressions are restricted to appear in at most two bordering regions (i.e., first and second quadrant, second and third quadrant, third and forth quadrant, and the first and fourth quadrant) of the face (Shreve et al., 2014).

Another popular facial segmentation method is splitting the face into a specific number (*m*×*n*) of blocks (Moilanen et al., 2014; Davison et al., 2015, 2016a; Wang et al., 2016a; Li et al., 2017). In the blocking representation, the motion changes in each block could by observed and analysis independently. However, with the increasing in the number of blocks (i.e., *m*×*n*), the computation load increases accordingly. Besides, features such as hairs and edges of face that appear in the blocks will affect the final feature vectors as these elements are not related to the facial motions.

A unique approach to facial segmentation for ME spotting is to split the face by Delaunay triangulation (Davison et al., 2016b). It gives more freedom to the shape that defines the regions of the face. Unfortunately, areas of the face that are not useful for ME analysis such as the cheek area may still be captured within the triangular regions. To address this problem, more recent methods partition the face into a few region-of-interests (ROIs) (Polikovsky et al., 2009; Polikovsky and Kameda, 2013; Liong et al., 2015, 2016b,c, 2018; Davison et al., 2016b; Li et al., 2018). The ROIs are regions that correspond to one or more FACS action units (AUs). As such, these regions contain rigid facial motions when the muscles (AUs) are activated. Some studies (Liong et al., 2015, 2016b,c; Davison et al., 2016b) show that ROIs are more effective compared to the use of the entire face in constraining the salient locations for spotting.

### 3.2. Spotting

Facial micro-expression spotting, or “*micro-movement”* spotting [a term coined by Davison et al. (2016a)] refers to the problem of automatically detecting the temporal interval of a micro-movement in a sequence of video frames. Current approaches for spotting facial micro-movement can be broadly categorized into two groups: classifier-based methods (supervised/unsupervised) and rule-based (use of thresholds or heuristics) methods. There are many possible dichotomies; this survey discusses some early ideas, followed by two distinct groups of works – one on spotting ME movement or window of occurrence, another on spotting the ME apex. A summary of the existing techniques for spotting facial micro-expressions (or micro-movements) are depicted in Table 3.

**Table 3:** Facial micro-expression (or micro-movement) spotting works in literature.

**Work**	**Feature**	**Feature Analysis**	**Movement (M) / Apex (A)**	**Spotting technique**	**Database**
Polikovsky et al., 2009	3D gradient histogram	–		k mean cluster	High-speed ME database (not available)
Shreve et al., 2009	Optical strain	–	M	Threshold technique	USF
Wu et al., 2011	Gabor features	–	M	GentleSVM	METT (48 videos)
Shreve et al., 2011	Optical strain	–		Threshold technique	USF-HD
			M		Canal-9 (not available)
					Found videos (not available)
Polikovsky and Kameda, 2013	3D gradient histogram	–		k mean cluster	High-speed ME database (not available)
Shreve et al., 2014	Optical strain	–	M	Threshold technique	USF
					SMIC
Moilanen et al., 2014	LBP	✓		Threshold technique	CASME-A
			M		CASME-B
					SMIC-VIS-E
Davison et al., 2015	HOG	✓	M	Threshold technique	SAMM
Patel et al., 2015	Spatio-temporal integration of OF vectors	–	M	Threshold technique	SMIC-VIS-E
Liong et al., 2015	LBP correlation	–		Binary search	CASME II
	CLM		A		
	Optical strain				
Wang et al., 2016a	MDMD	✓	M	Threshold technique	CAS(ME)^2^
Xia et al., 2016	Geometrical motion	–	M	Random walk model	CASME
	deformation				SMIC
Liong et al., 2016b	LBP correlation	–	A	Binary search	CASME II
Liong et al., 2016c	LBP correlation	–	A	Binary search	CASME II
	Optical strain				
Davison et al., 2016a	HOG	✓	M	Threshold technique	SAMM
Davison et al., 2016b	3D HOG	✓		Threshold technique	SAMM
	LBP		M		CASME II
	OF				
Li et al., 2017	HOOF	✓		Threshold technique	CASME II
	LBP		M		SMIC-E-HS
					SMIC-E-VIS
					SMIC-E-NIR
Yan and Chen, 2017	LBP correlation	–		Peak detection	CASME II
	CLM		A		
	HOOF				
Ma et al., 2017	RHOOF	–	A	Threshold technique	CASME
					CASME II
Qu et al., 2017	LBP	✓	M	Threshold technique	CAS(ME)^2^
Duque et al., 2018	Riesz Pyramid	✓	M	Threshold technique	SMIC-E-HS
					CASME II

#### 3.2.1. Early works

In the early works by Polikovsky et al. (2009) and Polikovsky and Kameda (2013), 3D-HOG was adopted to extract the features from each of the regions in the ME videos. Then, *k*-means clustering was used to cluster the features to particular AUs within predefined facial cubes. “Spotting” was approached as a classification task: each frame is classified to neutral, onset, apex or offset, and compared with ground truth labels. The classification rates achieved were satisfactory, in the range of 68–80%. Although their method could potentially contribute to facial micro-movement spotting by locating the four standard phases described by FACS, there are two glaring drawbacks. First, their method was only tested on posed facial ME videos, which are not a good representation of spontaneous (naturally induced) facial MEs. Secondly, the experiment was run as a classification task in which the frames were clustered into one of the four phases; this is highly unsuitable for real-time spotting. The work of Wu et al. (2011) also treats the spotting task as a classification process. Their work uses Gabor filters and the GentleSVM classifier to evaluate the frames. From the resulting label of each frame, the duration of facial micro-expressions were measured according to the transition points and the video frame-rate. Subsequently, they are only considered as ME when their durations last for 1/25–1/5s. They achieved very high spotting performance on the METT training database (Ekman, 2003). However, this was not convincing on two counts; first, only 48 videos were used in the experiments, and second, the videos were synthesized by inserting a flash of micro-expression in the middle of a sequence of neutral face images. In real-world conditions, frame transitions would be much more dynamic compared to the abrupt changes that were artificially added.

Instead of treating the spotting task as frame-by-frame classification, the works of Shreve et al. (2009, 2011) are the first to consider the temporal relation from frame-to-frame and employ a threshold technique to locate spontaneous facial MEs. This follows a more objective method that does not require machine learning. Their works are also the first in the literature to attempt spotting both macro- (i.e., ordinary facial expressions) and micro-expressions from videos. In their work, optical strain, which represents the amount of deformation incurred during motion, was computed from selected facial regions. Then, the facial MEs are spotted by tracking the strain magnitudes across frames following these heuristics: (1) strain magnitude exceeds the threshold (calculated from the mean of each video) and is significantly larger than that of the surrounding frames, and (2) the duration of the detected peak can only last at most 1/5th of a second. A 74% true positive rate and 44% false positive rate was achieved in the spotting task. However, a portion of data used in their experiments were posed, while some of them (Canal-9 and Found Videos databases) were not published or are currently defunct. In their later work (Shreve et al., 2014), a peak detector was applied to locate sequences containing MEs based on strain maps. However, the details of the peak detector and threshold value were not disclosed.

#### 3.2.2. Movement spotting

Micro-expression movements can be located by identifying a "window" of occurrence, typically marked by a starting or *onset* frame, and an ending or *offset* frame. In the work by Moilanen et al. (2014), the facial motion changes were modeled by feature difference (FD) analysis of appearance-based features (i.e., LBP) that incorporates the Chi-Square (χ^2^) distance to form the FD magnitudes. Only the top 1/3 of total blocks (per frame) with the greatest FD values were chosen and averaged to an initial feature value representing the frame. The contrasting difference vector is then computed to find relevant peaks from across the sequence. Spotted peak frames (i.e., the peaks that exceed the threshold) are compared with the provided ground truth frames; and considered true positive if they fall within the span of *k*/2 frames (where *k* is half of the interval frames in the window) before the onset and after the offset. The proposed technique was tested on CASME-A, CASME-B, and SMIC-VIS-E, achieving a true positive rate of 52, 66, and 71%, respectively.

The same spotting approach was adopted by Li et al. (2017) and tested on various spontaneous facial ME databases: CASME II, SMIC-E-HS, SMIC-E-VIS, and SMIC-E-NIR. This work also indicated that LBP consistently outperforms HOOF in all the datasets with higher AUC (area-under-the-ROC-curve) values and lower false positive rates. To spot facial micro-expressions on the new CAS(ME)^2^ database, the same spotting approach (Moilanen et al., 2014) is adopted by Wang et al. (2016a). Using their proposed main directional optical flow (MDMD) approach, ME spotting performance on the CAS(ME)^2^ is 0.32, 0.35, and 0.33 for recall, precision and F1-score, respectively. For all these works (Moilanen et al., 2014; Wang et al., 2016a; Li et al., 2017; Qu et al., 2017), the threshold value for peak detection is set by taking the difference between the mean and max value of the contrasting difference vector and multiplying it by a fraction in the range of [0,1]. Finally, this value is added with the mean value of the contrasting difference vector to denote the threshold. By these calculations, at least one peak will always be detected as the threshold value will never exceed the maximum value of the contrasting difference vector. This could potentially result in misclassification of non-ME movements since it will *always* detect a peak. Besides, pre-defining the ME window intervals (which obtains the FD values) may not augur well with videos captured at different frame rates. To address the potentiality of a false peak, these works (Moilanen et al., 2014; Davison et al., 2015; Wang et al., 2016a; Li et al., 2017; Qu et al., 2017) proposed to compute the baseline threshold based on a neutral video sequence from each individual subject in the datasets.

In the work of Davison et al. (2015), all detected sequences which are less than 100 frames are denoted as true positives, in which eye blinks and eye gaze are included; while peaks that are detected but not coded as a movement are classed to false positives. The approach achieved scores of 0.84, 0.70, and 0.76 for recall, precision, and F1-measure, respectively on the SAMM database. In their later works, Davison et al. (2016a) and Davison et al. (2016b) introduced “individualized baselines,” which are computed by taking a neutral video sequence for the participants and using the χ^2^ distance to get an initial feature for the baseline sequence. The maximum value of this baseline feature is identified as the threshold. This improved their previous attempt by a good margin.

A number of innovative approaches were proposed. Patel et al. (2015) computed optical flow vectors over small local regions and integrated them into spatiotemporal regions to find the onset and offset times. In another approach, Xia et al. (2016) applied random walk model to compute the probability of frames containing MEs by considering the geometrical deformation correlation between frames in a temporal window. Duque et al. (2018) designed a system that is able to differentiate between MEs and eye movements by analyzing the phase variations between frames based on the Riesz Pyramid.

#### 3.2.3. Apex spotting

Besides spotting facial micro-movements, a few other works focused on spotting a specific type of ME phase, particularly the *apex* frame (Liong et al., 2015, 2016b,c; Yan and Chen, 2017). The apex frame, which is the instant indicating the most expressive emotional state in an ME sequence, is believed to be able to effectively reveal the true expression for the particular video. In the work by Yan and Chen (2017), the frame that has the largest feature magnitude was selected as the apex frame. A few interesting findings were revealed: CLM (which provides geometric features) is especially sensitive to contour-based changes such as eyebrow movement, and LBP (which produces appearance features) is more suitable for detecting changes in appearance such as pressing of lips; however, OF is the most all-rounded feature as it is able to spot the apex based on the resultant direction and movement of facial motions. A binary search method was proposed by Liong et al. (2015) to automatically locate the apex frame in a video sequence. By observing that the apex frames are more likely to appear in areas concentrated with peaks, the proposed binary search method iteratively partitions the sequence into two halves, by selecting the half that contains a higher sum of feature difference values. This is repeated until a single peak is left. The proposed method reported a mean absolute error (MAE) of 13.55 frames and standard error (SE) of 0.79 on CASME II using LBP difference features. A recent work by Ma et al. (2017) used Region HOOF (RHOOF) based on 5 regions of interests (ROIs) for apex detection, which resulted in more robust results.

### 3.3. Performance metrics

The ME spotting task is akin to a binary detection task (ME is present/not present), hence typical performance metrics can be used. Moilanen et al. (2014) encouraged the use of a Receiver Operating Characteristic (ROC) curve, which was adopted in most subsequent works (Patel et al., 2015; Xia et al., 2016; Li et al., 2017). In essence, the spotted peaks, which are obtained based on a threshold level, will be compared against ground truth labels to determine whether they are true or false spots. If one spotted peak is located within the frame range of [onset - $\frac{N-1}{4}$, offset + $\frac{N-1}{4}$] of a labeled ME clip, the spotted sequence (*N* frames centered at the peak) will be considered as a true positive ME; otherwise the *N* frames of spotted sequence will be counted as false positive frames. The specified range considers a tolerance interval of 0.5 s, which corresponds to the presumed maximum duration of MEs. To obtain the ROC curve, true positive rate (TPR), and false positive rate (FPR) are computed as follows:

(1) $$\begin{array}{rcl} \text{TPR} & = & \frac{\text{Number of frames of correctly spotted MEs}}{\text{Total number of ground truth ME frames from all samples}} \end{array}$$

(2) $$\begin{array}{rcl} \text{FPR} & = & \frac{\text{Number of incorrectly spotted frames}}{\text{Total number of non-ME frames from all samples}} \end{array}$$

Recently, Tran et al. (2017) proposed a micro-expression spotting benchmark (MESB) to standardize the performance evaluation of the spotting task. Using a sliding window based multi-scale evaluation and a series of protocols, they recognize the need for a fairer and more comprehensive method of assessment. Taking a leaf out of object detection, the Intersection over Union (IoU) of the detection set and ground truth set was proposed to determine if a sampled sub-sequence window is positive or negative for ME (threshold set at 0.5).

Several works that focused on the spotting of the apex frame (Yan et al., 2014b; Liong et al., 2015, 2016b,c) used Mean Absolute Error (MAE) to compute how close are the estimated apex frames to the ground-truth apex frames:

(3) $$\begin{array}{r} \text{MAE}=\frac{1}{N}\overset{N}{\underset{i\;=\;1}{\sum }}|e_{i}| \end{array}$$

When spotting is performed on the raw long videos, Liong et al. (2016c) introduced another measure called Apex Spotting Rate (ASR), which calculates the success rate in spotting apex frames within the given onset and offset range of a long video. An apex frame is scored 1 if it is located between the onset and offset frames, and 0 otherwise:

(4) $$\begin{array}{l} \text{ASR}=\frac{1}{N}\sum_{i\;=\;1}^{N}\delta_{i}\\ \text{where }\delta =\{\begin{array}{ll} 1, & \text{if }f^{*}\in (f_{i,\text{onset}},f_{i,\text{offset}})\\ 0, & \text{otherwise} \end{array} \end{array}$$

## 4. Recognition of facial micro-expressions

ME recognition is a task that classifies an ME video into one of the universal emotion classes (e.g., Happiness, Sadness, Surprise, Anger, Contempt, Fear, and Disgust). However, due to difficulties in the elicitation of micro-expressions, not all classes are available in the existing datasets. Typically, the emotion classes of the collected samples are unevenly distributed; some are easier to elicit hence they have more samples collected.

Technically, a recognition task involves feature extraction and classification. However, a pre-processing stage could be involved prior to the feature extraction to enhance the availability of descriptive information to be captured by descriptors. In this section, all the aforementioned steps are discussed.

### 4.1. Pre-processing

A number of fundamental pre-processes such as face landmark detection and tracking, face registration and face region retrieval, have all been discussed in section 3 for the spotting task. Most recognition works employ similar techniques as those used for spotting, i.e., ASM (Milborrow and Nicolls, 2014), DRMF (Asthana et al., 2013), Face++ (Megvii, 2013) for landmark detection; LWM (Goshtasby, 1988) for face registration. Meanwhile, division of the facial area into regions is a step often found within various feature representation techniques (discussed in section 4.2) to further localize features that change subtly. Aside from these known pre-processes, two essential pre-processing techniques have been instrumental in conditioning ME data for the purpose of recognition. We discuss these two steps which involve *magnification* and *interpolation* of ME data.

The uniqueness of facial micro-expressions is in its subtleness, which is one of reasons why recognizing them automatically is very challenging. As the intensity levels of facial ME movements are very low, it is extremely difficult to discriminate ME types among themselves. One solution to this problem is to exaggerate or magnify these facial micro-movements. In recent works (Park et al., 2015; Zarezadeh and Rezaeian, 2016; Li et al., 2017; Wang et al., 2017), the Eulerian Motion Magnification (EMM) (Wu et al., 2012) method was employed to magnify the subtle motions in the ME videos. The EMM method extracts the frequency bands of interest from the different spatial frequency bands obtained from the decomposition of an input video, by using band-pass filters; these extracted bandpass signals at different spatial level are amplified by a magnification factor α to magnify the motions. Li et al. (2017) demonstrated that the EMM method helps to enlarge the difference between different categories of micro-expressions (i.e., inter-class difference); thus the recognition rate is increased. However, larger amplification factors may cause undesirable amplified noise (i.e., motions that are not induced by MEs), which may degrade recognition performance. To prevent over-magnifying ME samples, Le Ngo et al. (2016a) theoretically estimated the upper bounds of effective magnification factors. Besides, the authors also compared the performance of the amplitude-based Eulerian motion magnification (A-EMM) and phase-based Eulerian motion magnification (P-EMM); with the To deal with the distinctive temporal characteristic of different ME classes, a magnification scheme was proposed by Park et al. (2015) to adaptively select the most discriminative frequency band needed for EMM to magnify subtle facial motions. A recent work by Le Ngo et al. (2018) showed that Global Lagrangian Motion Magnification (GLMM) can contribute toward better recognition capability compared to local Eulerian based approaches, particularly at higher magnification factors.

Another concern for ME recognition is with the uneven length (or duration) of ME video samples. In fact, it can contribute to two contrasting scenarios: (a) the case of short duration videos, which restricts the application of the feature extraction techniques which require varied temporal window size (e.g., LBP-based methods that can form binary patterns from varied radius); and (b) the case of long duration videos, whereby redundant or replicated frames (due to high frame rate capture) could deteriorate the recognition performance. To solve the problem, the temporal interpolation method (TIM) is applied to either up-sample (clips that are too short) or down-sample (clips that are too long) clips to produce clips of similar frame lengths.

Briefly, TIM takes original frames as input data to construct a manifold of facial expressions; then it samples on the manifolds for a particular number of output frames (refer to Zhou et al., 2011 for detailed explanation). It is shown by Li et al. (2017) that modifying the frame length of ME videos can improve the recognition performance if the number of interpolated frames are small. However, when the interpolated frames are increased, the recognition performance is somewhat hampered due to over-interpolation. Therefore, the appropriate interpolation of the ME sequence is vital in preparation for recognition. An alternative technique Sparsity-Promoting Dynamic Mode Decomposition (DMDSP) (Jovanović et al., 2014) was adopted by Le Ngo et al. (2015) and Le Ngo et al. (2016b) to select only significant dynamics in MEs to form sparse structures. From the comprehensive experimental results shown in Le Ngo et al. (2016b), DMDSP achieved better recognition performance compared to TIM (on similar features and classifiers) due to its ability to keep only the significant temporal structures while eliminating irrelevant facial dynamics.

While the aforementioned pre-processing techniques showed positive results in improving ME recognition, yet these methods will notably lengthen the computation time of the overall recognition process. For a real-time system to be feasible, this cost has to be taken into consideration.

### 4.2. Representations

In the past few years, research in automatic ME analysis have been much focused on the problem of ME recognition: given an ME video sequence/clip, the purpose of recognition is to estimate its emotion label (or class). Table 4 summarizes the existing ME methods in the literature. From the perspective of feature representations, they can be roughly divided into two main categories: *single-level* approaches and *multi-level* approaches. Single-level approaches refer to frameworks that directly extract feature representations from the video sequences; while for multi-layer approaches, the image sequences are first transformed into another domain or subspace prior to feature representation to exploit other kinds of information to describe MEs.

**Table 4:** Benchmarking facial micro-expression recognition works in literature.

**Papers**	**Pre-processing**	**Features**	**Classifier**	**Accuracy (%)**		**F1-score (%)**	
				**CASME II**	**SMIC**	**CASME II**	**SMIC**
**LOSO**							
Li et al., 2013	–	LBP-TOP	SVM	–	48.78	–	–
Liong et al., 2016a	–	OSF + OS and weighted LBP-TOP	SVM	–	52.44	–	–
Liong et al., 2014a	–	OS	SVM	–	53.56	–	–
Liong et al., 2014b	–	OS weighted LBP-TOP	SVM	42.00	53.66	0.38	0.54
Le Ngo et al., 2014	–	STM	Adaboost	43.78	44.34	0.3337	0.4731
Wang et al., 2015b	–	LBP-MOP	SVM	44.13	50.61	–	–
Xu et al., 2017	–	Facial Dynamics Map	SVM	45.93	54.88	0.4053	0.538
Oh et al., 2016	–	Monogenic + LBP-TOP	SVM	–	–	0.41	0.44
Oh et al., 2015	–	Riesz wavelet + LBP-TOP	SVM	–	–	0.43	–
Liong et al., 2018	ROIs	LBP-TOP	SVM	46.00	54.00	0.32	0.52
Wang et al., 2014b	–	LBP-SIP	SVM	46.56	44.51	0.448	0.4492
Le Ngo et al., 2016a	A-EMM	LBP-TOP	SVM	–	–	0.51	–
Le Ngo et al., 2016b	DMDSP	LBP-TOP	SVM	49.00	58.00	0.51	0.60
Park et al., 2015	Adaptive MM	LBP-TOP	SVM	51.91	–	–	–
Happy and Routray, 2017	–	HFOFO	SVM	56.64	51.83	0.5248	0.5243
Liong et al., 2016b	–	Bi-WOOF	SVM	–	–	0.56	0.53
Huang et al., 2016	–	STCLQP	SVM	58.39	64.02	0.5836	0.6381
Huang et al., 2015	–	STLBP-IP	SVM	59.51	57.93	0.57^*^	0.58^*^
Liong et al., 2016c	–	Bi-WOOF (apex frame)	SVM	–	–	0.61	0.62
He et al., 2017	–	MMFL	SVM	59.81	63.15	–	–
Kim et al., 2016	–	CNN + LSTM	Softmax	60.98	–	–	–
Liong and Wong, 2017	–	Bi-WOOF + Phase	SVM	62.55	68.29	0.65	0.67
Zheng et al., 2016	–	LBP-TOP	RK-SVD	63.25		–	–
Zong et al., 2018a	–	Hierarchical STLBP-IP	KGSL	63.83	60.78	0.6110	0.6126
Huang and Zhao, 2017	TIM	STRBP	SVM	64.37	60.98	–	–
Huang et al., 2017	–	Discriminative STLBP-IP	SVM	64.78	63.41	–	–
Allaert et al., 2017	–	OF Maps	SVM	65.35	–	–	–
Li et al., 2017	TIM+EVM	HIGO	SVM	67.21	68.29	–	–
Zheng, 2017 ^†‡^	–	2DSGR	SRC	–	71.19	–	–
Liu et al., 2016 ^†^	–	MDMO	SVM	67.37	80.00	–	–
Davison et al., 2017 ^‡^	–	HOOF	SVM	76.60	–	0.55	–
**LOVO**							
Wang et al., 2015a ^†^^‡^	TIM	LBP-TOP on TICS	SVM	62.30	–	–	–
Yan et al., 2014a	–	LBP-TOP	SVM	63.41	–	–	–
Wang et al., 2014a	TIM	DLSTD	SVM	63.41	68.29	–	–
Happy and Routray, 2017	–	HFOFO	SVM	64.06	56.1	0.6025	0.5536
Liong et al., 2014a	–	OS weighted LBP-TOP	SVM	65.59	–	–	–
Wang et al., 2015b	–	LBP-MOP	SVM	66.8	60.98	–	–
Wang et al., 2014b	–	LBP-SIP	SVM	67.21	–	–	–
Ping et al., 2016		LBP-TOP	GSLSR	67.89	70.12	–	–
Park et al., 2015	Adaptive MM	LBP-TOP	SVM	69.63	–	–	–
Wang et al., 2017	EVM	LBP-TOP	SVM	75.3	–	–	–
Li et al., 2017	TIM+EVM	HIGO	SVM	78.14	75	–	–
**OTHER PROTOCOLS**							
Zhang et al., 2017	–	LBP-TOP and HOOF	RF	62.5	–	-–	–
*Evenly Distributed*							
Jia et al., 2017	–	SVD+ LBP/LBP-TOP	KNN	65.5	–	–	–
*Random Test (20 times)*							
Peng et al., 2017 ^§^^‡^	–	DTSCNN	SVM	66.67	–	–	–
*three-fold cross-validation*							
Adegun and Vadapalli, 2016 ^†^	–	LBP-TOP	ELM	96.12	–	–	–
*five-fold cross-validation*							

† *Not all the samples in the dataset were used in the experiments*.

‡ *Different number of emotion classes were used in the experiments*.

§ *Combined CASME I/II database was used*.

* *Not reported in paper, but computed from confusion table provided*.

Feature representation is a transformation of raw input data to a succinct form; typically in face processing, representations can be from two distinct categories: geometric-based or appearance-based (Zeng et al., 2009). Specifically, geometric-based features describe the face geometry such as the shapes and locations of facial landmarks; whereas appearance-based features describe intensity and textural information such as wrinkles, furrows, and other patterns that are caused by emotion. However from previous studies in facial expression recognition (Fasel and Luettin, 2003; Zeng et al., 2009), it is observed that appearance-based features are better than geometric-based features in coping with illumination changes and mis-alignment error. Geometric-based features might not be as stable as appearance-based features as they need precise landmark detection and alignment procedures. For these similar reasons, appearance-based feature representations have become more popular in the literature on ME recognition

#### 4.2.1. LBP-based methods

Among appearance-based feature extraction methods, local binary pattern on three orthogonal planes (LBP-TOP) is widely applied in many works (Li et al., 2013; Guo et al., 2014; Le Ngo et al., 2014, 2015, 2016a,b; Yan et al., 2014a; Adegun and Vadapalli, 2016; Zheng et al., 2016; Wang et al., 2017). Most existing datasets (SMIC, CASME II, SAMM) have all reported the LBP-TOP as their baseline evaluation method. LBP-TOP is an extension of its low-level representation, local binary pattern (LBP) (Ojala et al., 2002), which describes local texture variation along a circular region with binary codes which are then encoded into a histogram. LBP-TOP extracts features from local spatio-temporal neighborhoods over three planes: the spatial (XY) plane similarly to the regular LBP, the vertical spatio-temporal (YT) plane and the horizontal spatio-temporal (XT) plane; this enables LBP-TOP to dynamically encode temporal variations.

Subsequently, several variants of LBP-TOP were proposed for the ME recognition task. Wang et al. (2014b) derived Local Binary Pattern— Six Interception Points (LBP-SIP) from LBP-TOP by considering only the 6 unique points lying on three intersecting lines of the three orthogonal planes as neighbor points for constructing the binary patterns. By reducing redundant information from LBP-TOP, LBP-SIP reported better performance than LBP-TOP in this task. A more compact variant, LBP-MOP (Wang et al., 2015b) was constructed by concatenating the LBP features from only three mean images, which are the temporal pooling result of the image stacks along the three orthogonal planes. The performance of LBP-MOP was comparable to LBP-SIP, but with its computation time dramatically reduced. While LBP considers only pixel intensities, spatio-temporal completed local quantized patterns (STCLQP) (Huang et al., 2016) exploited more information containing sign, magnitude, and orientation components. To address the sparseness problem (in most LBP variants), specific codebooks were designed to reduce the number of possible codes to achieve better compactness.

Recent works have yielded some interesting advances. Huang and Zhao (2017) proposed a new binary pattern variant called spatio-temporal local Radon binary pattern (STRBP) that uses Radon transform to obtain robust shape features. Ben et al. (2017) proposed an alternative binary descriptor called Hot Wheel Patterns (HWP) (and its spatio-temporal extension HWP-TOP) to encode the discriminative features of both macro- and micro-expressions images. A coupled metric learning algorithm is then used to model the shared features between micro- and macro-expression information.

#### 4.2.2. Optical flow-based methods

As suggested in several studies (e.g., Li et al., 2017), the temporal dynamics that reside along the video sequences are found to be essential in improving the performance of ME recognition. As such, optical flow (OF) (Horn and Schunck, 1981) based techniques, which measure the spatio-temporal changes in intensity, came into contention as well.

In the work by Xu et al. (2017), a proposal to extract only principal directions of the OF maps was purportedly to eliminate abnormal OF vectors that resulted from noise or illumination changes. A similar concept of exploiting OF in the main direction was employed by Liu et al. (2016) to design main directional mean optical flow (MDMO) features. MDMO is a ROI-based OF feature, which considers both local statistic (i.e., the mean of OF vectors in the bin with the maximum count in each ROI) and its spatial location (i.e., the ROI to which it belongs). Unlike the aforementioned works which exploited only the single dominant direction of OF in each facial region, Allaert et al. (2017) determined the consistent facial motion, which could be in multiple directions from a single facial region. The assumption was made based on the fact that facial motions spread progressively due to skin elasticity, hence only the directions that are coherent in the neighboring facial regions are extracted to construct a consistent OF map representation.

Motivated by the use of optical strain (OS) for ME spotting (Shreve et al., 2009, 2014), Liong et al. (2014a) proposed to leverage on its strengths for ME recognition. OS is derived from OF by computing the normal and shear strain tensor components of the OF. This enables the capture of small and subtle facial deformation. In their work, the OS magnitude images are temporally pooled to form a single pooled OS map; then the resulting map is max-normalized and resized to a fixed smaller resolution before transforming into a feature vector that represent the video. To emphasize the importance of active regions, the authors (Liong et al., 2014b) proposed to weight local LBP-TOP features with different weights which were generated from the temporal mean-pooled OS map. This allows regions that actively exhibit MEs to be given more significance, hence increasing the discrimination between emotion types. In a more recent attempt, Liong et al. (2016b) proposed a Bi-Weighted Oriented Optical Flow (BI-WOOF) descriptor which applies two schemes to weight the HOOF descriptor locally and globally. Locally, the magnitude components were used to weight the orientation bins within each ROI; the resultant locally weighted histograms are then weighted again (globally) by multiplying with the mean optical strain (OS) magnitude of each ROI. Intuitively, a larger change in the pixel's movement or deformation will contribute toward a more discriminative histogram. Instead of considering the whole image sequences, the authors also demonstrated promising recognition performance using only two frames (i.e., the onset frame and the apex frame) instead of using whole sequences. This was able to reduce the processing time by a large margin.

Zhang et al. (2017) proposed to aggregate the histogram of the oriented optical flow (HOOF) (Chaudhry et al., 2009) with LBP-TOP features region-by-region to generate local statistical features. In their work, they revealed that fusing of local features within each ROI can capture more detailed and representative information than globally done. In the work by Happy and Routray (2017), fuzzy histogram of optical flow orientation (FHOFO) was proposed for ME recognition. In HFOFO, the histograms are only the collection of orientations without being weighted by the optical flow magnitudes; the assumption was made that MEs are so subtle that the induced magnitudes should be ignored. They also introduced a fuzzification process that considers the contribution of an orientation angle to its surrounding bins based on fuzzy membership functions; as such smooth histograms for motion vector are created.

#### 4.2.3. Other methods

Aside from methods based on low-level features, there are also numerous techniques proposed to extract other types of feature representations. Lu et al. (2014) proposed a Delaunay-based temporal coding model (DTCM) to encode the local temporal variation (in grayscale values) in each subregion obtained by Delaunay triangulation and preserve the ones with high saliency as features. In the work of Li et al. (2017), the histogram of image gradient orientation (HIGO), which is a degenerate variant of HOG, was employed in the recognition task. It uses simple vote rather than weighted vote when counting the responses of the gradient orientations. As such, it could depress the influence of illumination contrast by ignoring the magnitude. The use of color space was also experimented in the work of Wang et al. (2015a), where LBP-TOP features were extracted from Tensor Independent Color Space (TICS). In TICS, the three color components (R, G, and B) were transformed into three uncorrelated components which are as independent as possible to avoid redundancy and thus increase the recognition performance. The Sparse Tensor Canonical Correlation Analysis (STCCA) representation proposed by Wang et al. (2016b) offers a solution to mitigate the sparsity of spatial and temporal information in a ME sequence.

Signal components such as magnitude, phase and orientation can be exploited as features for ME recognition. Oh et al. (2015) proposed a monogenic Riesz wavelet framework, where the decomposed magnitude, phase, and orientation components (which represent energy, structural and geometric information respectively) are concatenated to describe MEs. In their extended work (Oh et al., 2016), higher-order Riesz transform was adopted to exploit the intrinsic two-dimensional (i2D) local structures such as corners, junctions, and other complex contours. They demonstrated that i2D structures are better representative parts than i1D structures (i.e., simple structures such as lines and straight edges) in describing MEs. By supplementing the robust Bi-WOOF descriptor (Liong et al., 2016b) with Riesz monogenic phase information derived from the onset-apex difference image (Liong and Wong, 2017), recognition performance can be further boosted.

Integral projections are an easy way of simplifying spatial data to obtain shape information along different directions. The LBP-Integral Projection (IP) technique proposed by Huang et al. (2015) applies the LBP operator on these projections. A difference image is first computed from successive frames (to remove face identity) before it is projected into two parts: vertical projection and horizontal projection. This method was found to be more effective than directly using features derived from the original appearance information. In their extended work (Huang et al., 2017), original pixel information is replaced by extracted subtle emotion information as input for generating spatio-temporal local binary pattern with revisited integral projection (STLBP-RIP) features. To further enhance the discriminative power of these features, only features with the smallest Laplacian scores are selected as the final feature representation.

A few works increase the significance of features by means of excluding irrelevant information such as pose and subject identity, which may obstruct salient emotion information. Robust principal component analysis (RPCA) (Wright et al., 2009) was adopted in Wang et al. (2014a) and Huang et al. (2016) to extract subtle emotion information for feature extraction. In Wang et al. (2014a), the extracted subtle emotion information was encoded by local spatio-temporal directional (LSTD) to extract more detailed spatio-temporal directional changes on the *x, y*, and *t* directions from each plane (XY, XT, and YT). Lee et al. (2017) proposed an interesting use of Multimodal Discriminant Analysis (MMDA) to orthogonally decompose a sample into three modes or “identity traits” (emotion, gender and race) in a simultaneous manner. Only the essential emotion components are magnified before the samples are synthesized and reconstructed.

Recently, numerous new works have begun exploring other forms of representation and mechanisms. He et al. (2017) proposed a strategy to extract low-level features from small regions (or cubes) of a video by learning a set of class-specific feature mappings. Jia et al. (2017) devised a macro-to-micro transformation model based on singular value decomposition (SVD) to recognize MEs by utilizing macro-expressions as part of the training data. This overcomes the lack of labeled data in MEs databases. There were various recent attempts at casting the recognition task as one arising from a different problem. Zheng (2017) formulated it as a sparse approximation problem and presented the 2D Gabor filter and sparse representation (2DSGR) technique for feature extraction. Zhu et al. (2018) drew inspiration from similarities between MEs and speech to propose a transfer learning method that projects both domain signals to a common subspace. In a radical move, Davison et al. (2017) proposed to re-group MEs based on Action Units (AUs) instead of by emotion categories, which are arguably susceptible to bias in self-reports used during the construction of dataset. Their experimental results on CASME II and SAMM showed that recognition performance should be higher than what is currently expected from other works that used emotion labels.

### 4.3. Classification

The last stage in an ME recognition task involves the classification of the emotion type. Various types of classifiers have been used for the task of ME recognition such as *k*-Nearest Neighbor (*k*-NN), support vector machine (SVM), random forest (RF), sparse representation classifier (SRC), Relaxed K-SVD, group sparse learning (GSL) and extreme learning machine (ELM). From the literature, the most widely used classifier is the SVM. SVMs are computational algorithms that construct a hyperplane or a set of hyperplanes in a high or infinite dimensional space (Cortes and Vapnik, 1995). During the training of SVM, the margins between the borders of different classes are sought to be maximal. Compared to other classifiers, SVMs are robust, accurate, and very effective even in cases where the number of training samples is small. On the contrary, two other notable classifiers—RF and *k*-NN are seldom used in the ME recognition task. Although the RF is generally quicker than SVM, it is prone to overfit when dealing with noisy data. The *k*-NN uses an instance-based learning process which may not be suitable for sparse high-dimensional data such as face data.

To deal with the sparseness of MEs, several works tried using relaxed K-SVD, SRC, and GSL techniques for classification. However, each of these methods tackle the sparseness of MEs differently. The relaxed K-SVD (Zheng et al., 2016) learns a sparse dictionary to distinguish different MEs by minimizing the variance of sparse coefficients. The SRC (Yang et al., 2012) used in Zheng (2017) represents a given test sample as a sparse linear combination of all training samples; hence the sparse nonzero representation coefficients are likely to concentrate on training samples that are of the same class as the test sample. A Kernelized GSL (Zong et al., 2018a) is proposed to facilitate the process of learning a set of importance weights from hierarchical spatiotemporal descriptors that can aid the selection of the important blocks from various facial blocks. Neural networks can offer a one-shot process (feature extraction and classification), with a remarkable ability to extract complex patterns from data. However, a substantial amount of labeled data is required to properly train a neutral network without overfitting it, resulting in it being less favorable for ME recognition since labeled data is limited. The ELM (Huang et al., 2006), which is naturally just feed-forward network with a single hidden layer was used by Adegun and Vadapalli (2016) to classify MEs.

### 4.4. Experimental protocol and performance metrics

The original dataset papers (Li et al., 2013; Yan et al., 2014a; Davison et al., 2016a) all propose the adoption of the Leave-One-Subject-Out (also known as “LOSO”) cross-validation as the default experimental protocol. This is done with consideration that the samples were collected by eliciting the emotions from a number of different participants (i.e., *S* number of subjects). As such, cross validation should be carried out by withholding a particular subject *s* while the other *S*−1 subjects are used in the training step. This removes the potential identity bias that may arise during the learning process; a subject that is being evaluated could have been seen and learned in the training step. A number of other works used the Leave-One-Video-Out (“LOVO”) cross-validation protocol instead, which exhaustively divides all samples into *S* number of possible train-test partitions. This protocol is deemed to avoid irregular partitioning but is often likely to overestimate the performance of the classifier. A few works opted to report their results using their own choice of evaluation protocol, such as an evenly distributed sets (Zhang et al., 2017), random sampling of test partition (Jia et al., 2017), and five-fold cross validation (Adegun and Vadapalli, 2016). Generally, the works in literature can be categorized into these three groups, as shown in Table 4.

The ME recognition task reports the typical performance metric of *Accuracy*, which is commonly used in other image/video recognition problems. A majority of works in the literature report the Accuracy metric, which is simply the number of correctly classified video sequences over the total number of video sequences in the dataset. However, due to the imbalanced nature of the ME datasets which was first discussed by Le Ngo et al. (2014), Accuracy scores can be highly skewed toward classes that are larger as classifiers tend to learn poorly from classes that are less represented. Consequently, it makes more sense to report the *F1-Score* (or F-measure), which is the harmonic mean of the *Precision* and *Recall*:

(5) F1−Score = 2·Precision·RecallPrecision+Recall

(6) $$Precision\text{ =}\frac{tp}{tp+fp}$$

(7) $$Recall\text{ =}\frac{tp}{tp+fn}$$

where *tp*, *fp*, and *fn* are the number of true positives, false positives, false negatives, respectively. The overall performance of a method can be reported by *macro-averaging* across all classes (i.e., compute scores for each class, then average them), or by *micro-averaging* across all classes (i.e., summing up the individual *tp*, *fp*, and *fn* in the entire set before computing scores).

## 5. Challenges

The studies reviewed in sections 2, 3, and 4 show the progress in the research work in ME analysis. However, there is still considerable room for improvement in the performance of ME spotting and recognition. In this section, some recognized problems in existing databases and challenging issues in both tasks are discussed in detail.

### 5.1. Databases

Acquiring valuable spontaneous ME data and their ground truth is far from being solved. Among the various affective states, certain emotions (such as happiness) are relatively easier to be elicited compared to others (e.g., fear, sadness, anger) (Coan and Allen, 2007). Consequently, there is an imbalanced distribution of samples per emotion and number of samples per subject. This could be biased toward particular emotions that constitute a larger portion of the training set. To address this issue, a more effective way of eliciting affective MEs (especially to those are relatively difficult) should be discovered. Social psychology has suggested creative strategies for inducing affective expressions that are difficult to elicit (Coan and Allen, 2007). Some works have underlined the possibility of using other complementary information from the body region (Song et al., 2013) or instantaneous heart rate from skin variations (Gupta et al., 2018) to better analyze micro-expressions.

Almost all the existing datasets contain a majority of subjects from one particular country or ethnicity. Though it is common knowledge that basic facial expressions are universal across the cultural background, nevertheless subjects from different backgrounds may express differently toward the same elicitation, or at least with different intensity level as they may have different ways of expressing an emotion. Thus, a well-established database should comprise a diverse range of ethnic groups to provide better generalization for experiments.

Although much effort has been paid toward the collection of databases of spontaneous MEs, some databases (e.g., SMIC) lack important metadata such as FACS. It is generally accepted that human facial expression data need to be FACS coded. The main reason being that FACS AUs are objective descriptors and independent of subjective interpretation. Moreover, it is also essential to report the reliability measure of the inter-observers (or inter-coders) involved in the labeling of data.

Considering the implementation of real-life applications of ME recognition in the near future, existing databases that are constructed under studio environments, may not best represent MEs exhibited in real-life situations. Thus, developing and introducing real-world ME databases could bring about a leap of progress in this domain.

### 5.2. Spotting

Recent work on the spotting of MEs have achieved promising results on successfully locating the temporal dynamics of micro-movements; however, there is room for improvement as the problem of spotting MEs remains a challenging task to date.

#### 5.2.1. Landmark detection

Even though the facial landmark detection algorithms have made remarkable progress over the past decade, the available landmark detectors are not always accurate or steady. The unsteadiness of face alignment based on imprecise facial landmarks may result in significant noise (i.e., rigid head movements and eye gaze) associated with dynamic facial expressions. This in turn increases the difficulty in detecting the correct MEs. Thus, a more advanced robust facial landmark detection is required to correctly and precisely locate the landmark points on the face.

#### 5.2.2. Eyes: to keep or not keep?

To avoid the intrusion of eye blinks, majority of works in the literature simply mask out the eye regions. However, according to some findings (Zhao et al., 2011; Vaidya et al., 2014; Lu et al., 2015; Duan et al., 2016), the eye region is one of the most discriminative regions for affect recognition. As many spontaneous MEs involving muscles around eye regions, there is a need to differentiate between the eye blinks corresponding to certain expressions and those that are merely irrelevant facial motions. In addition, the onsets of the many MEs also temporally overlap with eye blinks (Li et al., 2017). Thus, this warrants a more robust approach at dealing with overlapping occurrences of facial motions.

#### 5.2.3. Feature-based or rule-based?

The few studies (Liong et al., 2015; Yan and Chen, 2017) investigated the effectiveness of individual feature descriptors in capturing the micro-movements for the ME spotting task. They have showed that micro-movements that are induced from different facial components actually resulted in motion changes from different perspectives such as appearance, geometric, and etc. For example, lifting up or down the eyebrows results in a clear contour change (geometrical), which could be effectively captured by geometric-based feature descriptors; pressing of lips could cause the variation in appearance but not the position, and thus appearance-based feature descriptors can capture these changes. Interestingly, they reported that motion-based features such as optical flow based features outperformed appearance-based and geometric-based features in the ME spotting. The problem remains that the assumptions made by optical flow methods are likely to be violated in unconstrained environments, rendering real-time implementation challenging.

Majority of existing efforts toward the spotting of MEs employ rule-based approaches that rely on thresholds. Frames with magnitude exceeding the pre-defined threshold value are the frames (i.e., the temporal dynamics) where ME appears. However, prior knowledge is required to set the appropriate threshold for distinguishing the relevant peaks from local magnitude variation and background noise. This is not really practical in the real-time domain. Instead, Liong et al. (2015) designed a simple divide-and-conquer strategy, which does not require a threshold to locate the temporal dynamics of MEs. Their method finds the apex frame based on a high concentration of peaks.

#### 5.2.4. Onset and offset detection

Further steps should also be considered to locate the onset and offset frames of these ME occurrences. While it is relatively easier to identify the peaks and valleys of facial movements, the onset and offset frames are much more difficult to determine. The task of locating the onset and offset frames will be even tougher when dealing with real-life situations where facial movements are continuously changing. Thus, the indicators and criteria for determining the onset and offset frames need to be properly defined and further studied. Spotting the ME onset and offset frames is a crucial step which can lead to automatic ME analysis.

### 5.3. Recognition

In the past few years, much effort has been done toward ME recognition, including developing new features to better describe MEs. However, due to the short elapsed duration and low intensity of MEs, there is still room for improvement toward achieving satisfactory accuracy rates. This could be due to several possible reasons.

#### 5.3.1. Block selection

In most works, block-based segmentation of a face to extract local information is a common practice. Existing efforts employed block-based segmentation of a face without considering the contribution from each of the blocks. Ideally, the contribution from all blocks should be varied, whereby the blocks containing the key facial components such as eyebrows, eyes, mouth, and cheek should be highlighted as the motion changes at these regions convey meaningful information from differentiating different MEs. Higher weights can be assigned to those regions that contain key facial components to enhance the discriminative power. Alternatively, the discriminative features from the facial blocks can be selected through a learning process; the recent work of Zong et al. (2018a) offers a solution to this issue.

#### 5.3.2. Type of features

Since the emergence of the ME recognition works, many different feature descriptors have been proposed for MEs. Due to the characteristic of the feature descriptors, the extracted features might carry different information (e.g., appearance, geometric, motion, etc). For macro-expressions, it has been shown in (Fasel and Luettin, 2003) and Zeng et al. (2009) that geometric-based features performed poorer than appearance- and motion-based features as they are highly dependent on the precision of facial landmark points. However, recent ME works (Huang et al., 2015, 2017) show that shape information is arguably more discriminative for identifying certain MEs. Perhaps different features may carry meaningful information for different expression types. This should be carefully exploited and taken into consideration during feature extraction process.

#### 5.3.3. Deep learning

The advancement of Deep Learning has prompted the community to look for new ways of extracting better features. However, a crucial ingredient to this remains as to the feasible amount of data necessary to train a model that does not over-fit easily; the small scale of data (lack of ME samples per category) and the imbalanced distribution of samples are the primary obstacles. Recently an approach by Patel et al. (2016) made an attempt to utilize deep features transferred from pretrained ImageNet models. The authors deemed that fine-tuning the network to the ME datasets is not plausible (insufficient data) and opted for a feature selection scheme. Some other works (Kim et al., 2016; Peng et al., 2017) have also begun exploring the use of deep neural networks by encoding spatial and temporal features learned from network architectures that are relatively “shallower” than those used in the ImageNet challenge (Russakovsky et al., 2015). This may be a promising research direction in terms of advancing the features used for this task.

#### 5.3.4. Cross-database recognition

Another on-going development that challenges existing experimental presuppositions is cross-database recognition. This setup mimics a realistic setting where training and test samples may come from different environments. Current recognition performance based on single databases, is expected to plunge under such circumstances. Zong et al. (2017, 2018b) proposed a domain regeneration (DR) framework, which aims to regenerate micro-expression samples from source and target databases. The authors aptly point out that much is still to be done to discover more robust algorithms that work well across varying domains. The first ever Micro-Expression Grand Challenge (Yap et al., 2018) was held with special attention given to the importance of cross-database recognition settings. Two protocols – Hold-out Database Evaluation (HDE) and Composite Database Evaluation (CDE), were proposed in the challenge, using the CASME II and SAMM databases. The reported performances (Khor et al., 2018; Merghani et al., 2018; Peng et al., 2018) were poorer than most other works that apply only to single databases, indicating that future methods need to be more robust across domains.

### 5.4. Experiment related issues

#### 5.4.1. Evaluation protocol

An important issue that should be addressed in ME recognition is how the data is evaluated. Due to the different evaluation protocols used in existing works, a fair comparison among these works could not be adequately established. Currently, the two popular evaluation protocols that are widely applied in ME recognition are: leave-one-video-out cross-validation (LOVOCV) and leave-one subject-out cross validation (LOSOCV). The common *k*-fold cross-validation is not suitable as the current publicly available spontaneous ME datasets are highly imbalanced (Le Ngo et al., 2014). The number of samples per subject and the number of samples per emotion class in these datasets vary quite considerably. For instance, in the CASME II dataset, the number of samples that belong to the “Surprise” class is 25 compared to the 102 samples of the “Others” class; while the difference in the number of samples for “Subject 08” and “Subject 17” are 8 and 34, respectively. As such, with *k*-fold cross-validation, the fairness in evaluation is likely to be questionable. The same goes with employing LOVOCV, where only one video sample is left out as the test sample while the remaining samples are used for training; subsequently, the average accuracy across all folds is taken as the final result. This can possibly introduce additional biases on certain subjects that have more representation during the evaluation process. Moreover, the performance of such a protocol typically over-estimates the actual classifier performance due to a substantially large training set. It is paramount to stress that the LOSOCV protocol is a more convincing evaluation protocol as it separates the samples of the test set based on the subject identity. As such, the training model is not biased toward the identity of the subject (akin to face recognition instead). Naturally, this protocol also limits the ability of methods to learn the intrinsic micro-expression dynamics of each subject. The intensity and manner of which micro-expressions are shown may differ from person to person, hence compartmentalizing a subject altogether may inhibit the modeling process.

#### 5.4.2. Performance metrics

Besides the usage of evaluation protocol, the choice of performance metrics is also crucial to understanding the actual performance of automatic ME analysis. Currently, two performance metrics are used most widely: the Accuracy rate and F1-score. While the Accuracy rate is straightforward in calculation, it does not give an adequate reflection of the effectiveness of a classifier as it is susceptible to heavily skewed data (uneven distribution of samples per emotion class), a characteristic found in most current datasets. Also, the Accuracy rate merely shows the average “hit rate” across all classes; thus the performance of the classifier that deals with each emotion class is not revealed. It is a much preferred practice to report confusion matrices for better understanding of its per-class performances. From thereafter, performance metrics such as F1-score, Precision and Recall provide a better measure of a classifier's performance when dealing with imbalanced datasets (Sokolova and Lapalme, 2009; Le Ngo et al., 2014). The overall F1-score, Precision and Recall scores should be micro-averaged based on the total number of true positives, false positives, and false negatives.

#### 5.4.3. Emotion class

There are several existing works considering different number of emotion classes instead of using the emotion classes provided by the databases. For instance, in the works by Wang et al. (2015a) and Zheng (2017), the authors considered only three or four emotion labels (i.e., Positive, Negative, Surprise, and/or Others) instead of the original emotion labels of the CASME II (i.e., Happiness, Surprise, Disgust, Repression, and Others). Due to the reduction in the number of emotion classes considered, the classification task could be relatively simpler compared to those that have more emotion classes. As a result, higher performances were reported but this also inhibits these works from fair benchmarking against other works on the merit of their methods. It is important to note also that the grouping of classes may be biased toward negative categories since there is only one positive category (Happiness).

Recently, Davison et al. (2017) challenged the current use of emotion classes by proposing the use of *objective classes*, which are determined by restructuring these new categories around the Action Units (AUs) that have been FACS coded. Samples from the two most recent FACS coded datasets, CASME II and SAMM, were re-grouped into these objective classes for their use. The authors argued that emotion classification requires the context of the situation for an interpreter to make a meaningful interpretation, while relying on self-reports (Yan et al., 2014a) can also cause further unpredictability and bias. Although FACS coding can objectively assign AUs to specific muscle movements of the face but the emotion type becomes less obvious. Lim and Goh (2017), through their fuzzy modeling, provided some insights as to why the emotional content in ME samples are non-mutually exclusive as they may contain traces of more than one emotion type.

## 6. Conclusion

Research on the machine analysis of facial MEs has witnessed substantial progress in the last few years as several new spontaneous facial MEs databases were made available to aid automatic analysis of MEs. This has spiked the interest of the affective and visual computing community with a good number of promising methods making headways in both automatic ME spotting and recognition tasks. This necessitates a comprehensive review of recent advances to better taxonomize the increasing number of existing works. In addition, this paper also summarizes the issues that have not received sufficient attention, but are crucial for feasible machine interpretation of MEs. Among the important issues that are yet to be addressed in the field of ME spotting:

- Handling macro movements: Differentiating between larger, macro facial movements such as eye blinks and twitches, for better spotting of the onset of MEs,
- Developing more precise spotting techniques that can cope with various head poses and camera views: Extension of current constrained environments toward more real-time “in-the-wild” settings will provide a major leap in practicality.
- Establishing a firm criteria for defining the onset and offset frames for MEs: This allows ME short sequences to be extracted from long videos, which in turn, can be classified into emotion classes.

For the ME recognition task, there are a few issues that deserve the community's attention:

- Excluding irrelevant facial information: As MEs are very subtle, it is a great challenge to remove other image perturbations caused by face alignment and slight head rotations which may interfere with the MEs.
- Improving feature representations: Encoding subtle movements are difficult even when feature representations are rich. This is due to limitations in the amount of data that is currently available.
- Encouraging cross-database evaluation: Evaluating within single databases often gives a false impression of a method's performance, especially when existing databases lack diversity.

## Author contributions

Y-HO and JS compiled and analyzed the works reviewed in this article, ACLN organized the structure of the review, and RCWP provided the critical analysis and necessary proof reading. All authors took part in the writing of the article.

### Conflict of interest statement

The authors declare that the research was conducted in the absence of any commercial or financial relationships that could be construed as a potential conflict of interest. The reviewer XL and handling Editor declared their shared affiliation.
